# Adaptation of a standardized lifestyle intervention to maximize health outcomes in adolescent metabolic and bariatric surgery patients

**DOI:** 10.1186/s12967-024-04953-x

**Published:** 2024-02-23

**Authors:** Maral Misserian, Alicia Wheelington, Rashon King, Jackson Francis, M. Sunil Mathew, Marlyn A. Allicock, Bethany R. Cartwright, Adejumoke Adewunmi, Aparajita Chandrasekhar, Dhatri Polavarapu, Faisal G. Qureshi, Sarah E. Barlow, Sarah E. Messiah

**Affiliations:** 1 School of Health Professions, University of Texas Southwestern Medical Center, Dallas, TX USA; 2 Children’s Health System of Texas, Dallas, TX USA; 3 Center for Pediatric Population Health, University of Texas Health Science Center at Houston (UTHealth) School of Public Health, 2777 North Stemmons Freeway, Suite 8400, Dallas, TX 75390 USA; 4 UTHealth School of Public Health at Houston, Dallas Campus, Dallas, TX USA; 5 Department of Pediatrics, University of Texas Southwestern Medical Center, Dallas, TX USA; 6 Touchstone Diabetes Center, University of Texas Southwestern Medical Center, Dallas, TX USA; 7 Department of Surgery, University of Texas Southwestern Medical Center, Dallas, TX USA; 8 Department of Pediatrics, UTHealth McGovern Medical School, Houston, TX USA

**Keywords:** Adolescent, Metabolic and bariatric surgery, Weight loss surgery, Lifestyle intervention, Weight loss

## Abstract

**Background:**

Metabolic and bariatric surgery (MBS) is safe and efficacious in treating adolescents with severe obesity. Behavioral/lifestyle programs can support successful preparation for surgery and post-MBS weight loss, but no standardized lifestyle intervention exists for adolescents. Here we describe the process of developing and adapting the Diabetes Prevention Program Group Lifestyle Balance (DPP/GLB) curriculum to support adolescents pre- and post-MBS.

**Methods:**

We collected both qualitative and quantitative data from a diverse group of adolescents (N = 19, mean age 15.2 years, range 13–17, 76% female, 42% non-Hispanic Black, 41% Hispanic, 17% other). Additionally, we included data from 13 parents, all of whom were mothers. These participants were recruited from an adolescent MBS program at Children’s Health System of Texas. In an online survey, we asked participants to rank their preferences and interests in DPP/GLB content topics. We complemented these results with in-depth interviews from a subset of 10 participants. This qualitative data triangulation informed the development of the TeenLYFT lifestyle intervention program, designed to support adolescents who were completing MBS and described here. This program was adapted from adolescent and parent DPP/GLB content preferences, incorporating the social cognitive model (SCM) and the socioecological model (SEM) constructs to better cater to the needs of adolescent MBS patients.

**Results:**

Adolescents’ top 3 ranked areas of content were: (1) steps to adopt better eating habits and healthier foods; (2) healthy ways to cope with stress; and (3) steps to stay motivated and manage self-defeating thoughts. Nearly all adolescent participants preferred online delivery of content (versus in-person). Mothers chose similar topics with the addition of information on eating healthy outside the home. Key themes from the adolescent qualitative interviews included familial support, body image and self-confidence, and comorbidities as key motivating factors in moving forward with MBS.

**Conclusions:**

The feedback provided by both adolescents and parents informed the development of TeenLYFT, an online support intervention for adolescent MBS candidates. The adapted program may reinforce healthy behaviors and by involving parents, help create a supportive environment, increasing the likelihood of sustained behavior change. Understanding adolescent/parent needs to support weight management may also help healthcare providers improve long-term health outcomes for this patient population.

## Background

Currently ~ 9% of 12- to 19-year-olds in the United States have severe obesity (body mass index or BMI at or above 120% of the 95th percentile adjusted for age and sex [1]). Severe obesity is associated with elevated blood pressure and lipids, type 2 diabetes, asthma, sleep apnea, liver disease, joint problems, and mental illness, including anxiety and depression [2]. Metabolic and Bariatric Surgery (MBS) is an effective, safe, and durable weight loss treatment option in adolescents with severe obesity [3–5]. According to the American Society for Metabolic and Bariatric Surgery (ASMBS), MBS is recommended for individuals with (1) a BMI ≥ 35 kg/m^2^, regardless of presence, absence, or severity of comorbidities; and (2) BMI of 30–34.9 kg/m^2^ and the presence of associated comorbidities [6]. In addition, the American Academy of Pediatrics (AAP) has endorsed MBS as safe for the pediatric population based on large prospective observational studies [7]. Recent analysis of the Metabolic and Bariatric Surgery Accreditation and Quality Improvement Program (MBSAQIP) has shown an increase in the use of MBS among youth in the United States [8].

In 2017, a National Institute of Health workshop titled “Developing Precision Medicine Approaches to the Treatment of Severe Obesity in Adolescents,” hosted by the National Institute of Diabetes and Digestive Kidney Disease (NIDDK), discussed opportunities to expedite research aimed at advancing precision medicine approaches for the treatment of severe obesity in adolescents. The NIDDK report from this workshop concluded that more research is needed to better understand the underlying etiology of severe pediatric obesity to develop effective intervention approaches, specifically calling attention to the need for more research dedicated to MBS in adolescents, and how behavioral lifestyle interventions can complement and support these youth [9]. While stand-alone behavioral lifestyle interventions have shown promise in pediatric populations with obesity, they are insufficient to achieve long-term clinically significant weight loss among adolescents with severe obesity [4, 10]. Despite the significant increases in the prevalence of adolescents with severe obesity, a systematic review of 36 published articles that included the combination of healthy lifestyle intervention and MBS among adolescents (1995–2019) showed no studies reporting outcomes or effect sizes for the combination or compound effects of lifestyle intervention in partnership with MBS. Moreover, only one study included a theoretical framework with key, testable constructs to support the combination of lifestyle and MBS interventions [11]. In a second systematic review examining the use of digital platforms to deliver behavioral lifestyle interventions to MBS patients [12], results showed that studies varied widely in design (qualitative to randomized controlled trials) and eHealth delivery method (telemedicine to blog post content) for pre- or post-MBS use. Further, no studies included adolescents, and very few reported (1) a conceptual framework to support study design/outcomes; and (2) race/ethnicity composition of participants.

**Fig. 1:**
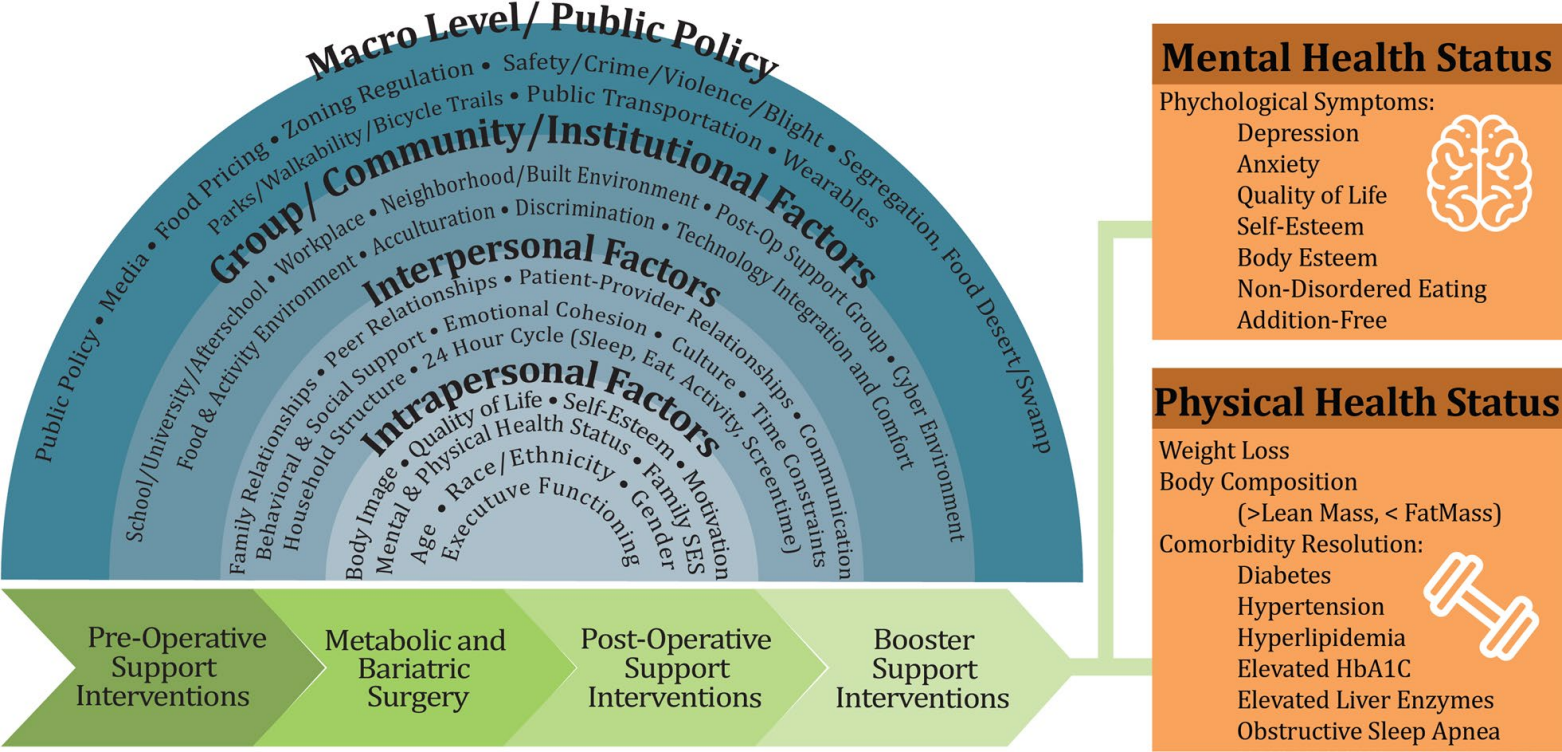
Socioecological model to support the adaption of a lifestyle support intervention to improve health outcomes for adolescents completing metabolic and bariatric surgery

Indeed, Ortiz et al. [13] recommended ecological approaches [14] to examine MBS outcomes among racial/ethnic minorities using mixed methods to understand how support systems influence postoperative adherence and health outcomes. The social-ecological model’s (SEM, Fig. 1) [15] four levels (intrapersonal, interpersonal, group/community, societal/environmental) are ideal as a framework to develop and adapt a pre- and post-MBS support intervention for the adolescent MBS population. Figure 1 provides examples explicit to our target population that are important to address to provide comprehensive support. We describe here the methods used to develop and adapt an MBS lifestyle support intervention for adolescent MBS patients.

## Methods

### Study purpose

**Fig. 2:**
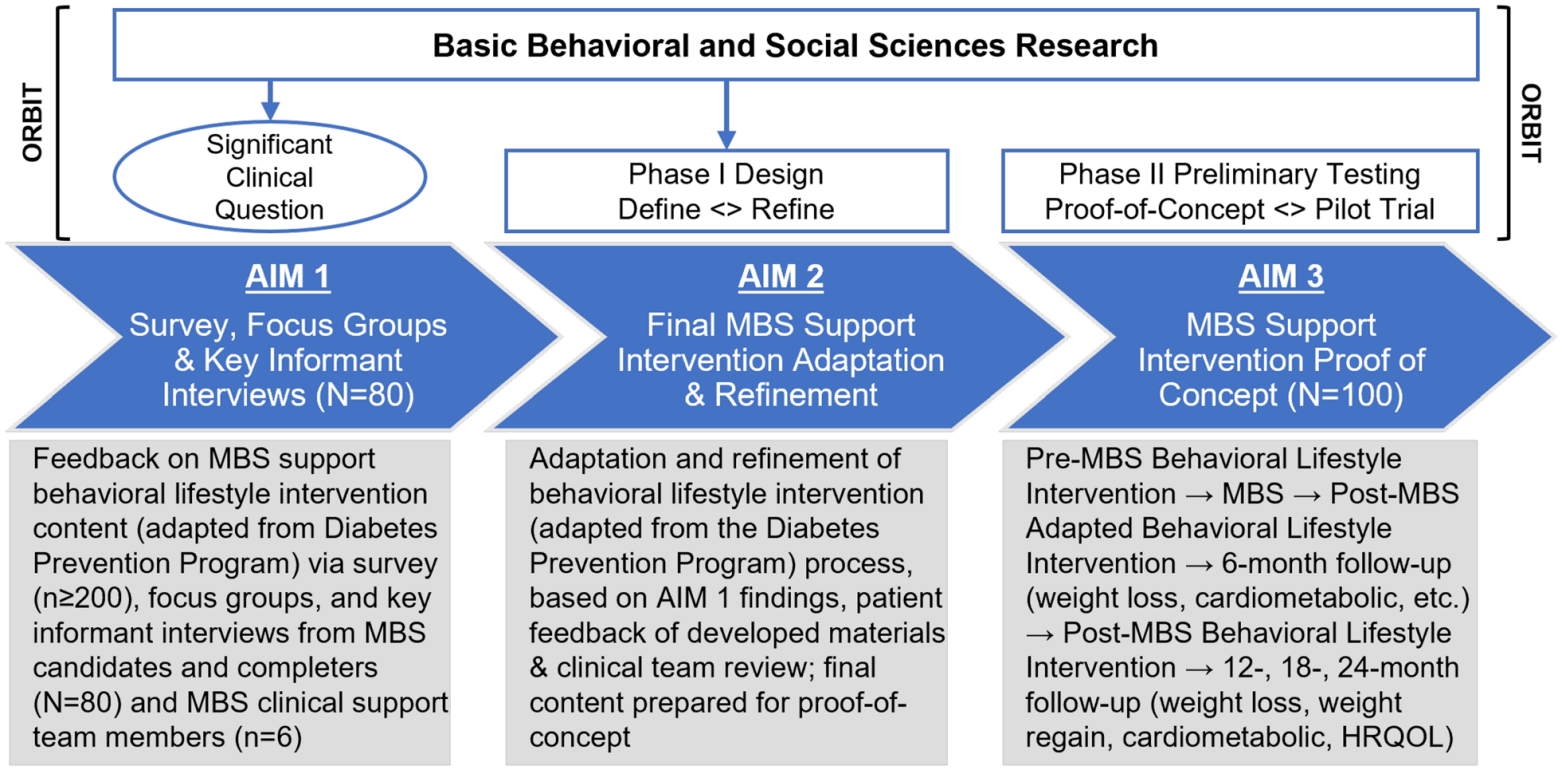
Study schematic adapted from the Obesity-Related Behavioral Intervention Trails (ORBIT) that focuses exclusively on the early, pre-efficacy phases of behavioral treatment development

Based on literature documenting the effectiveness of MBS for durable weight loss among adolescents, [16] the lack of existing lifestyle interventions to support youth pre-and post-MBS, and strong empirical support from the Diabetes Prevention Program in the prevention of diabetes in adults, we describe here the development of “TeenLYFT (lifestyle support for teens)”, completed by a collaborative team of experts in pediatric obesity and related fields (i.e., exercise physiology, nutrition, psychology) to expand and adapt the intervention for pre-post MBS support in youth. We describe the intervention adaptation process, which involves three phases. Our study flow is adapted from the Obesity-Related Behavioral Intervention Trials (ORBIT, Fig. 2) [17] systematic framework that focuses on the proof-of-concept phase of intervention development. ORBIT is used to guide efforts to translate basic behavioral science findings into behavioral treatments for preventing and treating chronic illness. The first phase took place over the course of 6 months and involved collecting mixed methods data via an online survey and in-depth interviews with parents and adolescents to inform the development and adoption of TeenLYFT. This coincided with ORBIT’s pre-phase I (Significant Clinical Question, Fig. 2). The second phase, which took place over an additional 6 months, focused on adapting and refining the information collected from adolescents and their parents. The third phase of the study, which coincides with ORBIT’s Phase II, is currently conducting a proof-of-concept trial of the adapted intervention among 20 adolescents who have completed MBS, and will not be discussed in this paper as it is in progress. Data were collected and managed using Research Electronic Data Capture (REDCap) web-based application hosted at Children’s Health System of Texas. The University of Texas Health System Institutional Review Board approved the study.

### Phase 1

The aim of Phase 1 was to generate quantitative and qualitative, or mixed methods data necessary to adapt the Diabetes Prevention Program Group Lifestyle Balance (DPP/GLB) [18] content, strategies, and delivery methods to explicitly support pre- and post-MBS, ethnically diverse (non-Hispanic white and black; Hispanic, other), adolescent patients and their families. This formative research included a survey and key informant interviews with adolescents, parents, and clinical team members. The survey and interviews consisted of ranking the priority of DPP/GLB information provided in the pre-post MBS support intervention, questions about method of delivery, and weight bias experiences. The interviews also focused on what content participants were interested in receiving pre-and post-operatively to support their weight loss. Below we describe the DPP/GLB content all participants were asked to rank in terms of preference in the survey on and discuss in an in-depth qualitative interview.

### Diabetes Prevention Program Group Lifestyle Balance (DPP/GLB) description [18]

The Diabetes Prevention Program Group Lifestyle Balance (DPP/GLB) was developed by the University of Pittsburgh. Its goal is to prevent or delay the development of the comorbidities associated with obesity, prediabetes, and diabetes by providing tools and education to adults who have overweight, prediabetes, or diabetes to help them achieve a healthy weight and lifestyle. This is based on the principles of self-monitoring and using mindfulness and problem-solving skills to work toward specific goals. The DPP/GLB program contains 22 sessions including topics supporting nutrition, exercise, motivation, and stress management education and skills. Table 1 [19] summarizes the topics covered. These sessions are delivered in a group setting over one year. Sessions 1 to 12 are the core sessions, and they are delivered once a week over 4 months. The core sessions are designed to provide the basic education. Sessions 12 to 16 are transition sessions, and they are delivered once every 2 weeks over 2 months. The transition sessions reinforce the core sessions and encourage the participants to practice their behavior change skills independently. Sessions 17 to 22 are support sessions, and they are delivered once a month over 6 months. In the support sessions, the core diabetes and cardiovascular health messages are further discussed and are devoted to describing how skills learned in the previous session are or were practiced over the last month, and to review new topics or skills.

**Table 1:** Summary of diabetes prevention/program group lifestyle balance [18] sessions and content

Session number	Topic
Core sessions	
Session 1	Welcome to the Diabetes Prevention Program Group Lifestyle Balance (DPP/GLB) Gives an overview of the DPP/GLB program including setting a 7% weight loss goal
Session 2	Be a calorie detective This session teaches participants how to monitor their calories and their fat intake
Session 3	Healthy eating This session contains tips to include healthy foods in the daily routine, such as eating more fruits and vegetables, lean meats, and healthy fats while staying away from calorie-dense foods containing added sugars and unhealthy fats
Session 4	Move those muscles Content includes setting physical activity goals, providing exercises for different muscle groups, and reviewing exercise safety guidelines
Session 5	Tip the calorie balance This session is about balancing calories and how it is related to weight loss. It also provides tips on balancing a daily menu to keep caloric intake on track
Session 6	Take charge of what’s around you This session teaches participants how to develop positive activity and food cues. Those include recognizing hunger and fullness and having gym clothes and shoes nearby to engage in physical activity
Session 7	Problem solving This session provides five steps to problem solving, including weighing the pros and cons of a situation and choosing to stay on track with a weight loss plan
Session 8	Step up your physical activity This session focuses on staying active and safely increasing the intensity and time of an activity
Session 9	Manage slips and self-defeating thoughts This session emphasizes staying away from negative thoughts and replacing them with positive ones
Session 10	Four keys to healthy eating out This session provides tips on how to make healthier choices in restaurants
Session 11	Make social cues work for you This session includes how to turn social cues that might disrupt the weight loss journey into positive cues
Session 12	Ways to stay motivated This session discusses stress management and stresses the importance of staying motivated to reach the weight loss journey
Transition sessions	
Session 13	Strengthen your physical activity plan This session includes the benefits of resistance training and stresses the importance of stretching after each exercise session. It also explains how to increase weight and repetitions as one becomes proficient with the present routine
Session 14	Take charge of your lifestyle Even though the DPP/GLB meetings are now once a month, this session is about helping the participants to consistently monitor their weight and to stay on track with their physical activity
Session 15	Mindful eating, mindful movement This session teaches the participants the techniques to eat and move mindfully
Session 16	Manage your stress This session gives the opportunity to participants to talk about their stress levels. It also provides them with strategies to manage their stress
Support sessions	
Session 17	Sit less for your health This session reviews with the participants the time they spend sitting and provides them with suggestions on how to reduce their sitting time
Session 18	More volume, fewer calories This session discusses how to add volume to a meal without adding too many calories. It also provides some recipes that are easy to make yet add volume to the meal. As a result, one might feel full and eat less food
Session 20	Balance your thoughts This session is meant to help participants reflect on how changing their lifestyle helped them reach their weight loss goal. Furthermore, it encourages participants to eliminate negative thoughts and replace them with positive ones
Session 21	Heart health This session is all about heart disease, its causes, and prevention strategies. It also educates participants about blood cholesterol and how proper nutrition can support healthy levels
Session 22	Look back and look forward As this is the last session, participants are encouraged to continue with the healthy habits they have acquired throughout the past year. They are also advised to plan for eating and physical activity in the next 3–6 months

*DPP/GLB Procedures*. [19] In the first session, each participant receives a binder with material for the first session. At each subsequent session, participants receive material for that session for the binder. Session one introduces the participants to the DPP/GLB program and asks them to reflect on the reasons why they are taking part in this program. In addition, all participants set a weight loss goal of 7% and at least 150 min of physical activity (PA) per week. Each participant records his or her goal and is provided a weight and PA activity tracking sheet in the DPP/GLB material. Participants are encouraged to weigh themselves once a week. Each session starts with the presentation of the topic covered and then participants are asked to fill up a worksheet related to the topic. The teacher makes sure that the worksheets are being completed and then goes over the worksheet questions in a discussion format. At the end of each session there is a to-do list that is completed at home by each participant. For example, if the session subject is calorie tracking, then participants must track the calories they are eating by measuring their food and record the number of calories eaten per day in their binder. These to-do lists are checked at the beginning of each class to ensure compliance.

MBS patients often have prediabetes or diabetes [2] and many adolescent patients will transition into adulthood soon after surgery; therefore, the DPP/GLB is appropriate for pre- and post-MBS lifestyle changes and has been proven as an effective program for diabetes management in adults with excess weight. The current study aimed to determine what aspects of the established DPP/GLB needed to be adapted to support teens [17].

### Survey procedures

#### Study inclusion/exclusion criteria

Participants must have met the following inclusion criteria to complete the survey and qualitative interview; (a) Be between 12 and 18 years; (b) must meet National Institutes of Health criteria to qualify for MBS for adolescents (BMI > 35 kg/m^2^ and at least one existing co-morbidity [e.g. elevated blood pressure, hypercholesterolemia, etc.] or a BMI > 40 kg/m^2^) and (c) Consents/assents to participate in the study [17]. Conversely, exclusion criteria included the following; (a) Does not meet National Institutes of Health (BMI > 35 kg/m^2^ and at least one existing co-morbidity [e.g. elevated blood pressure, hypercholesterolemia, etc.] or a BMI > 40 kg/m^2^); (b) Is type 1 diabetic or type 2 insulin dependent diabetic; and/or (c) Refuses to participate in the study [17].

*Participants.* A total of 19 participants (76% females, 41% Hispanic, 42% non-Hispanic Black, 17% other, mean age 15.2 years) preparing for MBS were recruited from the Children's Health System of Texas in Dallas. Following parental and adolescent consent, the adolescents completed a brief online survey. This survey encompassed inquiries about demographics, exercise habits, utilization of weight loss support tools, preferred types of support tools to assist with their pre and post MBS weight loss journey, DPP/GLB content preferences, and desired platforms for receiving these support tools.

#### Survey results

*Adolescent Summary Results*. Adolescents ranked the following as their top three preferred areas of content interest: (1) steps to adopt better eating habits and healthier foods; (2) healthy ways to cope with stress; and (3) steps to stay motivated and manage self-defeating thoughts. Nearly all adolescent participants preferred online delivery of content versus in-person.

*Parent Summary Results.* Mothers chose similar topics with the addition of information on eating healthy outside the home.

While presentation of all qualitative analysis is beyond the scope of this paper, key themes identified in analysis from the adolescent interviews included familial support, body image and self-confidence, and comorbidities as key motivating factors in moving forward with MBS.

### Phase 2

The aim of phase 2 was to adapt the DPP/GLB content described above, as informed by AIM 1 qualitative and quantitative data, to support pre-and post-MBS among racially and ethnically diverse adolescent patients and their families. Below we describe this process in detail.

### TeenLYFT adaptation

“TeenLYFT” [17] is an ongoing study conducted at Children’s Health System of Texas. It has three aims described in Fig. 2. For AIM 2 which involves the adaptation of DPP/GLB, based on our AIM 1 findings, we have developed an adolescent MBS-focused intervention by applying the SEM and Social Cognitive Theory (SCT) (Fig. 1) [15] constructs which are shown in Table 2. The SCT explains behavior in terms of personal factors and the environment interacting constantly in a way that a change in one area has implications for the others. Its constructs include observational learning skills and monitoring as well as the development of self-regulation.

**Table 2:** DPP/GLB content by the socioecological model (SEM) and social cognitive theory (SCT) constructs, examples of adaptation for the TeenLYFT metabolic and bariatric surgery support intervention for adolescents

SEM constructs	Intrapersonal level	Interpersonal level	Group/Community level	Macro level
SCT constructs	Personal factors		Environmental influences	
	Behavior (Change)			
Observational learning	Intro to safe exercising; learning to read food labels; new nutrition intake	Share relatable stories & information about lifestyle change and challenges; transformation weight loss stories	Community-based lifestyle programs; local events to prevent post-MBS weight regain; cooking classes: easy, affordable recipes	PCPs referrals to DPP; PCP involvement in pre-post MBS support
Reinforcement	Goal setting for sustained weight loss; healthy eating; exercise; create a healthy relationship with food	Supervised physical activity sessions; rewards for successful completion, and for adding more fruits and vegetables; incentives group session participation	Community-based DPPs; physical activity incentives (reduced gym fees); grocery discounts	Calorie and macro/micronutrient content skills; app to support exercise skills
Self-control	Self-monitoring of micro/macro nutrient content to prevent weight regain; regulating exercise habits	Active listening; life coach discussions; MBS team nutritionist; social support network; supporting exercise	Social/group DPP/GLB MBS support groups in community; weight wellness programs (WWP) and other networks	Food label mastery; restaurant menu calorie mastery; exercise mastery
Self-efficacy	Weekly goals	Family meal planning	Fitness classes	Restaurant menus
Knowledge	Evaluation of self-esteem/body image; motivation; weight & health goals; Health-related quality of life (HRQOL); Exercise goals	Communication; best practices; in diet, activity, sleep & tech (MyFitnessPal); benefits of support	Assessment of school, home, cyber, neighborhood resources for prolonged support	Crime/safety; food swamps; public transportation knowledge mastery
Skill building	Body acceptance; self-confidence, mindfulness; goal setting (to support weight loss retention); Managing setbacks	Sleep hygiene tracking; food records; setting reminders; exercise tracking; social support strategies (group meetings)	Wellness resources at school & in the community; social support groups to overcome weight loss hurdles; use of social media support groups	Health/fitness trackers; walking maps; journaling; safety/healthy information use (e.g., Shopwell app)

*Adaptation Procedures*. Over 6 months, the study team met multiple times weekly to adapt the original DPP/GLB curriculum modules as informed by the survey findings and qualitative data/analysis acquired from participants during Phase 1. The adapted DPP/GLB TeenLYFT intervention consists of four pre- and post-surgery modules based on the survey results from Table 3. The modules focused on (1) motivation, (2) healthy eating, (3) physical activity, and (4) stress management and were adapted based on standardized DPP/GLB content and survey and qualitative interview data and feedback (Table 4). Each module contains seven to eight videos ranging from one to three minutes except the physical activity module which contains 36 videos. The videos were created with the graphic design software Canva [20]. They are based on scripts provided by the registered dietitian and the psychologist on the team. Artificial intelligence (AI) voices were used to narrate the videos’ content.

**Table 3:** DPP/GLB Education Module Preference Survey Results, TeenLYFT participants

Education module	Priority
Eat well: Contains steps to adopt better eating habits and tips for choosing healthier foods	1 (tie)
Stay motivated: Offers steps to stay motivated and ideas for beating self-defeating thoughts	
Managing stress: Practice healthy ways to cope with stress	
Get active: Get suggestions for different physical activities based on your preferences	2
Take a fitness break practice: Quick exercises in movement and how to work these activities into your daily life	3 (tie)
Get back on track: Provides solutions, a step-by-step plan, and encouragement on your weight loss journey	
Commit to change: Contains testimonial videos and information about the program’s efficacy and is tailored to your age group	4
Activity tracking: Provides concrete steps that encourage you to track physical activity and options to meet weekly activity goals	5
Food tracking: Information about developing a food log and tracking the foods you eat	6
Eat well away from home: Helps with selecting healthy food choices away from home	7
When weight loss stalls: Helps you stay motivated in your weight loss journey	8
Check-in and keep going: Provides habits of others who have successfully reached their goals	9
Get support: Provides suggestions on how to find social support for positive changes	10

**Table 4:** Teen LYFT pre-metabolic and bariatric surgery content summary by module and weeks

Module topic	Description
Motivation (Week 1 or Session 1*)	Includes exercise and food tracking tips to eat healthy and lose the required weight to reach the surgery weight goal. Provides focus on reasons for change and strategies for commitment to goals over time
Physical activity (Week 2 or Session 2)	Includes a series of weight training and stretching exercises by muscle group. Has suggestions for planning exercise routines, ideas and tips for cardiovascular exercises, and exercise safety guidelines
Stress management (Week 3 or Session 3)	Includes strategies to manage stress such as basic relaxation skills, scripts for mindful eating, practical ways to avoid overeating and emotional eating, and finding alternatives to food when faced with stressful situations
Healthy eating (Week 4 or Session 4)	Includes tips for healthy eating, such as adding more fruits and vegetables to daily food consumption and choosing leaner meats, whole grains, and more nutritious snacks

*Pre-surgery modules are designed to guide and support adolescents with lifestyle change requirements ideally in the month to 2 months before the procedure. However, content was designed to accommodate these patient-level variations, emphasizing flexibility of delivery, and adaptability to individual circumstances, ensuring comprehensive support

*Cultural Adaptation Process.* To ensure content was culturally-adapted and relevant to our diverse participants, the following principles were used in the adaptation stage; (1) Peripheral, e.g., ensuring that relevant materials appeal to all participants such as exercises that are relevant to all adolescents; (2) Evidential e.g., using data and information relevant to target population (how we address the knowledge constructs) including ways to socialize with their adolescent peers; (3) Linguistic, e.g., ensuring the inclusion of commonly used words such as tracking, healthy, nutritious, mindful, tips, stress; and (4) Sociocultural, e.g., using concepts that reflect knowledge of the groups’ culture .that contain graphics from all cultures that were relevant to their background.

*Readability Assessment.* All content was designed for a 6th−grade reading level. Readability was assessed by Readability Calculations. [21]

*Delivery Procedures*. The four modules are delivered via Facebook and YouTube. The pre- and post-surgery videos are separated and each contain four modules. They are available to watch at any time. At the beginning of the Facebook page a guide is provided. It contains a suggestion of the order in which the videos are to be watched and the rules to follow while on the TeenLYFT Facebook page. A short quiz is available at the end of each module to test the participants’ knowledge. Participants can also like the videos or leave a comment. The same format is used to deliver the videos through YouTube. However, due to limitations within YouTube, participants are not provided a one-off quiz to fill out rather they are included in the framework of the videos.

*Pre surgery modules *(Table 4). Pre-surgery modules are designed to guide and support adolescents with lifestyle change requirements ideally in the month to 2 months before the procedure. However, we realize that this may vary by patient and the time necessary to complete all pre-operative medical and insurance clearances. Acknowledging the diverse timelines driven by individual patient needs, the TeenLYFT content was designed to accommodate these variations, emphasizing flexibility of delivery, and adaptability to individual circumstances, ensuring comprehensive support. The motivation module focuses on identifying reasons for pursuing MBS. It also includes tips to track exercise and food intake. Finally, it explains why tracking is important for increasing self-awareness and providing opportunities for self-reward. The physical activity module includes exercise safety rules and demonstrations of upper and lower body weight training exercises. It also includes some examples of cardiovascular exercises and ways to make physical activity a part of their daily routine. The stress management module describes mindful eating and the importance of eating slowly while enjoying food. It provides tips to avoid emotional eating such as removing junk food from sight and maintaining a regular eating schedule. The last module is the healthy eating module. It contains tips to incorporate different fruits and vegetables in their daily diet. It also has a list of healthy snacks to choose from. Content offered provides skills to stay on track with clinic preoperative requirements. Finally, it tackles the importance of eating healthy while away from home and provides tips to make healthy choices.

*Post-surgery modules *(Table 5). The post-surgery modules extend to 6 months after MBS is completed. The module topics are the same as the pre-surgery modules, but content is geared toward post-MBS guidelines [5]. Both the pre- and post-MBS content are aligned with topics covered in pre-and post-operative standard of care visits. Additional video content related to time-post-MBS guidelines is available every 2 weeks for 6 months. For example, in the first week after surgery, participants can only drink clear liquids, moving on slowly to full liquids at 4 weeks, soft foods around 6 weeks, and then full pieces of food after 6 to 8 weeks. They are also encouraged to start exercising a few days after surgery by walking, and then to move on to weight training, stretching and core exercises once cleared by their clinical surgery medical providers, often about 6 weeks after their surgery date [22]. The motivation and stress management modules support ongoing adherence to nutrition and physical activity recommendations by providing tips to manage stress and stay motivated across time. A short quiz is available for every 4 weeks, or for each set of eight videos, of content. Similar to the pre-surgery modules, participants can leave a comment or “like” each video. A survey is sent to the participants at 3- and 6-months post-surgery. In addition to the surveys, participants are contacted by one of the study team members to conduct 6-week and 6-month post-surgery interviews. The purpose of the surveys and interviews is to assess participant’s health and weight loss progress and get to collect feedback on perspectives about MBS, the education modules and their suggestions to improve any of the content. The post-surgery modules are summarized in Table 5. A sample of narration content from weeks one and two post-surgery is provided in Table 6**.**

**Table 5:** Teen LYFT post-metabolic and bariatric surgery content summary by module and weeks

Weeks	Modules			
	Motivation	Physical activity	Stress management	Healthy eating
1–2	Encourages self-confidence and a sense of achievement for completing surgery	Walking and stretching schedule for 2 weeks	New body and diet coping skills	Includes a list of clear and full liquids for the first two weeks after surgery
3–4	Tracking exercise and food daily and weight once a week	Ways to increase the time and intensity of exercise	Recommends planning fun and positive activities to boost mood and morale	A list of soft foods that can be eaten for the first two weeks after surgery
5–6	Tips to remove unhealthy food temptations from sight while increasing healthy options to avoid setbacks	A list of strength and cardiovascular exercises to follow safely	Introduces the mindful eating concept and provides tips to remove distractions such as TV, computer, and phone while eating	Reminder about the order that foods should be introduced: proteins, fruits and vegetables, and finally grains
7–8	Encourages celebration of achievements in dietary changes, increased activity, and mindfulness	Provides a sample of interval training exercises and encourages engagement in favorite sports	Promotes problem solving skills, understanding the problem, and finding solutions	Contains a list of healthy protein options to promote healing
9–10	Reminders to review goals before surgery and set new post-operative goals moving forward	Encouragement to stay consistent with exercise routines and increase amount and intensity safely	Provides some relaxation techniques to manage stress	Reminders to stay consistent with a healthy eating plan and avoid high-fat and processed foods
11–12	This is the 3-month mark. Encouragement to celebrate their success with their friends and family	Encouragement to be proud of their consistent engagement with physical activity to date	Teaches the importance of sleep and encourages planning ahead for meals when eating out	Review of the revision of starches and grains that can be consumed to ease digestion
13–14	Tips to manage slips and self-defeating thoughts	Includes another reminder to increase the intensity, duration, and perhaps type of exercises/activities	Includes tips to balance positive and negative thoughts to reduce stress levels	Contains more tips to consume easily digestible foods and avoid sugars and processed foods
15–16	Reviews activity tracking goals	Reiterates the importance of being consistent with an exercise plan and encourages weight training	Encourages body positivity	Tips to make healthier choices in restaurants and fast-food options
17–18	Teaches skills for establishing healthy habits and includes family support	Talks about the benefits of choosing a total body workout	Provides tips to cope with defeating thoughts and feelings	Emphasizes the importance of food pacing for overall success
19–20	Encouragement to revisit health and fitness goals	Includes tips to engage in mindful exercises such as yoga, meditation, tai chi, and breathing exercises	Encouragement to practice gratitude, be grateful for their life, experiences and acts of kindness from others	Encouragement to enjoy every food slowly and mindfully, removing all distractions
21–22	Provides ways to embrace progress and remember why they chose to complete surgery	Includes tips to stay active effortlessly, like walking to school instead of taking the bus, among others	Focuses on the importance of mindful eating to reduce stress and keep up with weight loss goals	Reminders that they are close to their 6-month clinic visit and revisits healthy eating tips
23–24	Reminders of their why (for personal health) and provides tips for their long-term success	Talks about endorphins (the good hormones) that exercise produces to promote happiness, encourages consistent movement	Emphasizes strengthening stress management skills as stress never goes away, but one can learn how to manage it	Praise all achievements to date in terms of eating healthier and sticking to a plan

**Table 6:** Week 1 and 2 teen LYFT post-metabolic and bariatric surgery content for adolescents

Module	Content
Motivation	On your way to *your why:* You’ve made it through your surgery process. The journey is just beginning. (‘CONGRATULATIONS, remember your ‘*why’,* you are on your way to achieving a healthier life. It’s up to you to make the choices that show *your why* to the world ‘Focus on your biggest goal.’ if it’s to enjoy time with family—get help with planning your health routine and see how they can support you. If it’s having more confidence, practice positive self-talk while you get ready for the day, or if you’re hoping to feel better in your body, be sure to get in plenty of walking at this time
Physical activity	Including exercise in your daily life after surgery is very important. You can start walking even 1 day after your surgery if you feel up to it. Remember to ask your doctor before engaging in any type of physical activity. Start walking for at least 10 to 15 min per day and increase your time by at least five minutes every day, Remember the FITT principle. “F” stands for frequency (how many times per day or week), “I” stands for intensity (how hard you are working). To simplify your intensity level, think on a scale of 1 to 10, 1 being very easy and 10 being very hard. The first “T” stands for time and the final “T” for the type of exercise that you are doing. Aim to walk every day. You can do walking intervals several times each day such as 10 min in the morning, 10 min after lunch and 10 min in the afternoon. You also want to include some core stability and stretching exercises in your routine. Stop exercising if you feel any severe nausea, shortness of breath, have cold sweats, lightheadedness or irregular pulse or palpitations. Sit or lie down and if the pain does not go away after 5 to 10 min, call your provider. If the pain does go away with time, tell your health care provider about this incident at your next clinic visit. In the case of chest pain or discomfort, stop exercising and rest. If it does not go away in 2 to 4 min call 9–11. If it does go away, tell your healthcare provider
Stress management	“You’ve really committed to a healthier life! Now that you are post-surgery it will be more important than ever to manage your stress. Sometimes, even if a person didn’t realize it, there’s a role that food plays in comforting or easing them in times of stress. Some of this is natural and you’ll be back to eating soft and solid foods soon, but right now following your medical recovery plan with liquids only is required. The surgery and recovery process can bring about stress, but remember—you have skills and you have practiced ways to handle stress that can be safe and healthy right now. Try using your skills—spend time outside, try breathing strategies, take time with others, create art, stay appropriately active and remember to spend 15–30 min at a time using preferred technology or enjoying your favorite down-time activities. Really practice these skills and allow your mind and your body to recover from this big step you’ve taken in your bariatric surgery journey.”
Healthy eating	On the 2nd week after your surgery, you can start drinking full liquids. Full liquids contain more calories. They include low fat milks, unsweetened milk alternatives such as soy, pea or high protein almond milk, other low fat dairy products, sugar free pudding, protein powders, premade protein shakes and breakfast powdered drinks with no sugar added dissolved in low fat milk or water. Try to keep a regular schedule, having a full liquid in the morning, in the early afternoon, and in the evening each day

## Discussion

The adaptation of the Diabetes Prevention Program Group Lifestyle Balance (DPP/GLB) to create TeenLYFT described here aimed to address the unique requirements and preferences of adolescent MBS patients. This adaptation process was guided by valuable mixed methods data from both patients and their families, as well as standardized preoperative MBS guidelines. In contrast to the original DPP/GLB content, which consisted of 22 in-person sessions over a span of one year, TeenLYFT streamlined this content into four key modules: motivation, healthy eating, physical activity, and stress management. This work addresses a significant gap in the healthcare landscape by providing a tailored and evidence-based lifestyle intervention for adolescent MBS patients. With the increased prevalence of severe obesity among young people, TeenLYFT can help support adolescents in achieving sustained weight management and overall health improvements.

The motivation module of DPP/GLB primarily focuses on elements such as goal setting, self-monitoring, and problem-solving skills. TeenLYFT integrated these elements with a clear recognition that motivation was a crucial area of interest for adolescent MBS patients. The program emphasized aligning personal motivations with specific health behavior goals. Patients are encouraged to reflect on their individual reasons for engaging with recommended behaviors and to continuously assess their progress towards these objectives. TeenLYFT videos include explicit examples of how to practice these skills, since staying motivated was indicated as a primary area of interest for participants. Low motivation or being uncertain about how to develop consistent behavioral compliance to lifestyle recommendations was a relevant theme when adolescents and their parents were asked about barriers to health behavior adherence in Phase 1. Consequently, this was a primary topic throughout our adaptation process in Phase 2. We prioritized individual patient values, goals, and reasons for engaging with and maintaining health compliance and this was embedded throughout our intervention videos which was consistent with the DPP/GLB content. For example, one of the DPP/GLB sessions is dedicated to reminding participants about the reasons why they joined the program and how to stay on track with their original weight loss goal. Similarly, in our adapted TeenLYFT program, the patients are asked to consider “their why '' or main reasons, at any given time, for engaging with healthy lifestyle recommendations, such as weekly weighing, regular exercise, or consistently eating a nutrient-dense meal plan. A constant focus on patient-driven motives for healthy behaviors was emphasized throughout all modules and videos. This was a way for patients to build motivation for using recommended tips and it provided an opportunity for patients to check-in with their current progress. Prioritizing personal motivation via “their why” offered a starting point for establishing new goals or staying committed to goals, even when perceived motivation, or feeling like doing necessary health behaviors, was limited.

Stress management was identified as a critical topic for adolescent MBS participants, and TeenLYFT took a distinct approach to address this concern. Unlike DPP/GLB, which dedicated a single module to stress management and another to mindfulness, TeenLYFT embedded stress management skills throughout all of its modules. This approach ensured that stress-related topics, including mindfulness, managing negative thought patterns, and avoiding emotional eating, were consistently addressed to provide comprehensive support. These included specific tips for mindfulness, or using present-focused attention, and managing unhelpful thinking and behavior patterns, such as negative self-talk, skipping meals or neglecting to track calories or exercise, and not getting adequate sleep. Although stress management was a module itself, we incorporated stress management skills within other modules (e.g., mindful eating and mindful movement in the healthy eating and physical activity modules; gratitude and recognizing progress in the motivation module).

Physical activity emerged as a top priority for participants in the pre-adaptation survey. In response, TeenLYFT introduced a new pre-surgery module that emphasized the importance of staying active and provided practical sample exercises. Similar to DPP/GLB, the videos included cardiovascular and strength training exercises, with a strong focus on proper form and safety. In the post-surgery modules, TeenLYFT guided patients through gradual activity increases as they recovered, [22] adhering to the FITT principle (frequency, intensity, time, type) and emphasizing the development of self-efficacy. The DPP/GLB aims to help participants develop exercise skills throughout the year and includes several sessions reinforcing the importance of physical activity. Engaging in consistent physical activity is one of the requirements of MBS, thus the adapted TeenLYFT videos serve as an exercise guide to advance patients toward that goal. Post-MBS physical activity content was designed to incrementally introduce activities as their surgical wounds start to heal and they become more comfortable with increased activity levels. Similar to the DPP/GLB layout, content changes every 2 weeks based on the FITT (frequency, intensity, time, type) principle. The videos not only contain exercise ideas, but every video includes tips to increase adolescent self-efficacy and confidence in their abilities to stay active.

Finally, healthy eating was another area of great interest for adolescent MBS participants. DPP/GLB includes sessions on topics such as reading food labels accurately, making healthy choices when dining out, and adopting healthy snacking habits. However, additional specialized dietary education is required for patients undergoing MBS. Food is digested differently post MBS, as the stomach is much smaller and cannot hold as much food as before. Thus, adolescents post MBS should eat a very limited amount of food at a time. Only clear liquids can be consumed the first week after surgery, followed by full liquids the second week. Following surgical recovery, they can start eating regular foods, but they are limited to a recommendation of 500–800 cal per day. It is not recommended to eat and drink at the same time since the stomach will fill up with liquids and will not be able to process additional solid food. In addition, patients must concentrate on foods containing a high density of protein and nutrients to support the body’s needs and ongoing healing process. Patients must also take multivitamins for life since they are unlikely to absorb enough from the foods that they are eating daily [23].

Tailored specifically for MBS patients, TeenLYFT reinforced the recommendations of the MBS clinic dietitian, including calorie tracking and the post-operative dietary requirements described above. The program also addressed portion sizes, healthy snack options, and strategies for making nutritious choices when eating outside the home. Specifically, videos reinforce the MBS clinic dietitian recommendations such as consuming and tracking daily calories and replacing one meal with a low-calorie, high protein shake post-operative. In addition, the clinic provides a handout including how to make healthier food choices as patients prepare for MBS. An extensive list of healthy food options in protein, carbohydrate and fat categories is part of the handouts. The videos also identify portion sizes and healthy snack options. In addition, they reinforce the importance of eating healthy even when out of the home environment and provide tips for healthy choices from restaurant menus. There is a new video every 2 weeks that covers the foods that participants can eat following MBS-specific guidelines. In addition to the motivation and stress modules, the healthy eating module reinforces their self-efficacy and self-management skills to lead them toward a successful weight loss journey long-term.

While adapting the DPP/GLB for TeenLYFT, several limitations were considered. The relatively small sample size of 19 adolescent participants may impact the generalizability of findings, and the availability of all pre and post-surgery intervention content at once could lead to non-linear consumption. To address these concerns, in a currently ongoing proof-of-concept study, TeenLYFT is conducting interviews and surveys at 6-weeks, 3- and 6-months post-MBS to collect valuable feedback and insights for the ongoing improvement of the program.

Overall, TeenLYFT represents a promising approach to supporting adolescent MBS patients, building upon the foundation of the DPP/GLB while tailoring it to meet their specific needs and preferences [17]. Future directions for this research will involve the continuous refinement of the TeenLYFT program based on ongoing feedback and evaluation, ultimately aiming for broader implementation in clinical settings. Additionally, exploring the potential integration of mobile applications and digital platforms to enhance accessibility and engagement among adolescent MBS patients could be a promising avenue for further development.

## Data Availability

All data/graphs adopted are available from corresponding authors upon proper request.

## Abbreviations

BMI: Body mass index
MBS: Metabolic bariatric surgery
ASMBS: American Society of Metabolic and Bariatric Surgery
AAP: American Association of Pediatrics
MBSAQIP: Metabolic and bariatric surgery accreditation and quality improvement program
NIDDK: National Institute of Diabetes and Digestive Kidney Disease
SEM: Social ecological model
SCM: Social cognitive model
DPP/GLB: Diabetes Prevention Program/Group Lifestyle Balance
PA: Physical activity
ORBIT: Obesity-Related Behavioral Intervention Trials
TeenLYFT: Lifestyle support for teens
